# Solubility of Biliary Calculi within the Gall-Bladder

**Published:** 1875-11

**Authors:** Ralph S. Goodwin

**Affiliations:** Thomaston, Conn.


### SOLUBILITY OF BILIARY CALCULI WITHIN THE GALL-BLADDER.

BY RALPH S. GOODWIN, M. D., OF THOMASTON, CONN.

In number 894 of the *Medical and Surgical Reporter*, published April 18th, 1874, appeared a communication from Dr. E. Burd, of Iowa, in which the writer expresses his belief that certain substances, such as Durande's mixture, which consists of sulphuric ether and turpentine, (three parts of the former to two of the latter,) as well as chloroform and ether used separately, when taken into the stomach, are capable of exerting a solvent action on biliary concretions already formed in the gall-bladder.

In support of this theory, the doctor relates the case of a patient who was evidently much benefited by taking Durande's mixture, claiming for it a solvent action on the concretion already formed, or in the process of formation, in the gall-bladder.

Not long since, in a paragraph coming from a London medical periodical, and going the rounds of the medical press in this country, it was claimed that a clergyman of England, who had suffered from gall-stone colic very many years, was finally permanently cured by the solvent action of chloroform taken persistently, in five-drop doses, *ter die.*

Now, these statements show that there is a fascination about this solvent theory, of the action of medicines on gall-stones, notwithstanding its absurdity, which it is difficult for the medical mind to resist.

I desire to say a few words in refutation of this theory, since I have had a little experience in cases of this kind.

A few years ago I had the fortune to encounter a very obstinate case of cholelithiasis, in which, contrary to the general rule, I had succeeded in catching upon a sieve, at different times, a number of the concrements, establishing the diagnosis. Not being at that time very certain as to the impossibility of the solution of gall-stones by medicines, and having failed by other measures to control the cholelithic diathesis, I determined to give the solvent medicines a trial.

I gave my patient, who was a woman of sixty years, fifteen drops of chloroform three times a day, and succeeded in inducing her to continue its use for three months, notwithstanding that during that time an attack of gall-stone colic occurred nearly every week. I then tried the succinate of iron, as recommended by Dr. T. H. Buckler, with the same object in view, but without satisfactory results; and finally I persuaded her to try the nauseating mixture of Durande, according to rule, for several months. This also was accompanied by no good results, except a larger collection of gall-stones with which to experiment; and so I became extremely skeptical as to the solvent power of medicines on biliary calculi. I then made a few simple experiments with the gall-stones which I had saved. I threw one of them into an ounce phial filled with chloroform, and though the specimen only weighed five grains, it required thirty-six hours to dissolve it completely.

Chloroform will dissolve a biliary calculus of any chemical variety, but it does not follow that it can be readily introduced into the blood in sufficient quantity to effect a solution of the concretion immersed in bile in the bottom of the gall-bladder, or lodged in a gall-duct.

Another experiment was this: I procured, on one occasion, a human gall-bladder half full of bile, having carried it away from an autopsy. I weighed one of my specimens of gall-stones, and dropped it into the bile contained in the bladder. I then dropped into the same thirty minims of pure chloroform, tied up the bladder, agitated it, and hung it in a secluded place. At the end of ten days I took out the specimen and weighed it. It had gained somewhat, rather than lost in weight, owing to its hygroscopic qualities. I then replaced it and added one drachm of pure chloroform. In fourteen days, I took it out and weighed it again, and found it had not lost weight. The chloroform had evaporated in both cases, leaving no odor behind. There was no appearance of erosions on the gall-stone. I had intended to try Durande's mixture in the same way, but was prevented by evidence of putrefaction in the bile.

Now, I reasoned thus: If a teaspoonful of chloroform, which is the greatest dose possible for any person to take, habitually, into the stomach, would not dissolve a gall-stone of very moderate dimensions, when dropped directly into a gall-bladder containing bile, outside of the human body, it would certainly be a wide stretch of fancy to expect it to do so when diluted through eighteen or twenty pounds of blood, and distributed all over the body, with a certainty that not more than one hundredth part would ever reach the vicinity of the gall-bladder. I do not claim, of course, that this experiment was entirely conclusive, since it was conducted outside of the body, and, therefore, the conditions were not the same as in the living body. But the conditions would be, theoretically, more favorable outside the body, as in the experiment, than during life. For we are not certain that the chloroform and ether undergo some sort of decomposition in the process of digestion, so that they do not appear in their full integrity in the presence of the gall-stone. It cannot be claimed that these substances are cumulative in the system, so that by repeated and prolonged exhibition they may finally exist in sufficient quantities to produce a solvent action. They are evanescent and volatile, and speedily find their way out of the blood by the kidneys, lungs, etc.

The same argument will hold in regard to Durande's remedy. In the year 1806, Thenard read, before the Academy of Science, at Paris, a paper, in which he showed the impossibility of the solution of gall-stones by Durande's method. I will quote from "Thudichum" (page 81) what he says on this subject:

"At a temperature of 32° R., the ether in the mixture must separate from the oil of turpentine, and evaporate. The mixture, moreover, could only be taken in moderate doses, and even when taken in large doses, no part of it could get into the gall-bladder, or, at least, so small a quantity that its solvent power could not be taken into account."

After expressing his disbelief in the solvent action of medicines on gall-stones, Mr. Thudichum himself says (page 86):

"I have used the mixture of Durande in some instances, and have always had some difficulty to prevent the patients from continuing it for an undue length of time; for I had found what I remembered to have seen recorded as the result of the experience of others, that when the mixture is used improperly or too long, or even according to rule, it is apt to cause inflammation. In one of my cases, where it was continued for years, the chronic inflammation of the liver, and the neighborhood of the gall-bladder, appeared to have been produced, or at least greatly aggravated, by this mixture, or by the turpentine which it contains."

Von Niemeyer says (Vol. I., p. 705) "the fact that ether and oil of turpentine dissolve biliary calculi placed in them does not justify the hope that they will dissolve any concrements in the gall-bladder, if they be introduced into the stomach. Hence, if Durande's remedy has a favorable influence on the conditions induced by gall-stones, as we must suppose it has, from the recommendations of numerous and good observers, this can only take place in some way which is entirely unknown to us."

Professor A. Flint, in his work on practice of medicine, (p. 438,) says: "It seems, however, absurd to suppose that these or other remedies can be introduced into the system, so as to enter into the composition of the bile largely enough to dissolve the cholesterin, of which mainly biliary calculi are composed; and it is evident that clinical proof of the success of remedies given for this end cannot be obtained, since, in general, the existence of calculi within the gall-bladder is not ascertained prior to their passage into the intestines."

Now, in the face of such authority and cogent reasoning as this, is it proper that we should still stick to the old theory of the solubility of gall-stones by medicine? And should we, in proof thereof, adduce cases from our practice which happened to get well in spite of the "dissolving treatment?"—*Medical and Surgical Reporter.*

